# Does the Coronectomy a Feasible and Safe Procedure to Avoid the Inferior Alveolar Nerve Injury during Third Molars Extractions? A Systematic Review

**DOI:** 10.3390/healthcare9060750

**Published:** 2021-06-18

**Authors:** Raphaela Capella de Souza Póvoa, Carlos Fernando de Almeida Barros Mourão, Thaise Cristina Geremias, Roberto Sacco, Ludmilla Silva Guimarães, Pietro Montemezzi, Angelo Cardarelli, Vittorio Moraschini, Mônica Diuana Calasans-Maia, Rafael Seabra Louro

**Affiliations:** 1 Graduate Program, Dentistry School, Universidade Federal Fluminense, Niteroi 24020-140, Brazil; raphaelacapella.ctbmf@gmail.com (R.C.d.S.P.); thaisecg@id.uff.br (T.C.G.); r.sacco@ucl.ac.uk (R.S.); ludmila_guimaraes62@hotmail.com (L.S.G.); 2 Biotechnology Department, Universidade Federal Fluminense, Niteroi 24020-140, Brazil; 3 Independent Researcher, 24128 Bergamo, Italy; m.montemezzi@libero.it; 4 Independent Researcher, 86170 Isernia, Italy; angelo_cardarelli@libero.it; 5 Periodontology Department, Dental Research Division, School of Dentistry, Veiga de Almeida University, Rio de Janeiro 20271-020, Brazil; vitt.mf@gmail.com; 6 Oral Surgery Department, Universidade Federal Fluminense, Niteroi 24020-140, Brazil; monicacalasansmaia@gmail.com

**Keywords:** coronectomy, intentional partial odontectomy, mandibular third molar, inferior alveolar nerve, wisdom tooth, nerve injury

## Abstract

This systematic review aimed to review the literature on the coronectomy technique and evaluate the incidence of success and complications as a surgical approach for inferior third molars. Online databases were searched for data on the frequency of inferior alveolar nerve damage, lingual nerve damage, root migration, pain, infection, dry socket, and extraction of the remaining root, and data on the necessity of reintervention were also extracted. Randomized clinical trials, controlled clinical trials, prospective cohort studies, and prospective and retrospective studies with or without the control group were intercepted. This systematic review was registered in PROSPERO (CDR 42020135485). Sixteen papers analyzed 2176 coronectomies in total, and only five of them were judged as appropriate according to methodological quality assessment. The incidence of inferior alveolar nerve injury was documented in 0.59% of the procedures, lingual nerve injury in 0.22%, infection 3.95%, dry socket 1.12%, extraction of the root 5.28%, and reintervention 1.13%. The pain was the most reported, in 22.04% of the population. This study provides an overview of the clinical success and complications of coronectomy, and their prevalence. A coronectomy may be considered a low-risk procedure and an option for treatment to avoid potential damage to nervous structures. However, patients should still undergo a full screening and evaluation of postsurgical procedures.

## 1. Introduction

The procedure of extraction of third molars accounts for from 35.9% to 58.7% of all oral surgical procedures, and is the most common procedure performed by oral maxillofacial surgeons [1,2,3]. The indications of the extraction of third molars according to the American Association of Oral and Maxillofacial Surgeons (AAOMS) are when the patient presents pain, ectopic position, tooth fracture, unrestorable caries, internal or external resorption of the tooth or adjacent teeth, tooth associated with cystic lesion or tumor, acute, chronic infection, or periodontal disease [4].

One of the possible complications, and the most common during the extraction of inferior third molars, is the IAN injury, with an incidence of from 0.5% to 8% [5,6,7,8]. The temporary paresthesia of the IAN ranges from 0.26% to 8.4%, with a permanent loss of more than 3.6%. Another injury that can occur is to the LN, which is less common, ranging from 0.1 to 22% [9]. The orthopantographic image can show the proximity of the third molar to the IAN. Rood and Shehab described seven signs that indicate the proximity of these structures: deviation or narrowing of the canal, periapical radiolucency, narrowing, darkening and curving of the roots, and loss of lamina dura over the wall of the mandibular canal all recorded a 30% incidence of injury [10]. When these radiographic markers are present, the indication for cone-beam-computed tomography (CBCT) is the gold standard, assisting in surgical procedure and avoiding damage to the IAN.

A new possibility of treatment for these cases of third molars with a high proximity to the IAN was first described in 1989 by Knutsson et al. [11]. The coronectomy consists of removing the crown, while leaving the roots intact, preventing injury to the IAN. Pogrel et al. described the sequence and basic rules needed to perform the technique in more detail: the crown and a sufficient part of the coronal portion of the roots must be removed until they are from 2 to 3 mm below the level of the alveolar crest [11,12,13]. There are few complications associated with the coronectomy, such as pain, postoperative infection, dry socket in the short-term, and healing delay and root migration in the long-term.

This study aims to review the literature on the coronectomy technique and evaluate the incidence of the success and complications of coronectomy as a surgical approach for inferior third molars. The evaluation of these complications was based on eight criteria: the frequency of inferior alveolar nerve damage, lingual nerve damage, root migration, pain, infection, dry socket, and extraction of the remaining root and necessity for reintervention. The selection of these criteria was chosen according to the frequency with which they are discussed in the literature.

## 2. Materials and Methods

### 2.1. Protocol and Registration

This SR followed the recommendations of the PRISMA guidelines [14]. The protocol for this SR was based on PRISMA-P [15] and registered in the PROSPERO database under the number CDR 42020135485. There were no deviations from the initial protocol.

### 2.2. Focused Question Based on PICOS Strategy

A systematic search of the literature was conducted to identify papers regarding the main question that was defined according to the population, intervention, comparison, outcome (PICO) format [16]. What are the clinical outcomes and complications (O) of third molar surgery coronectomy (I), with no comparison (C), in patients with the third molars suspect to be or in contact with the IAN, without any pathology associated (P)?

### 2.3. Outcome Measures

The primary outcome variable was IAN damage. The secondary outcomes were LN damage, root migration, pain, infection, dry socket, extraction of the remaining root, and the necessity for reintervention.

### 2.4. Search Strategy

The search was conducted from inception until June 2020, without other restrictions on dates or language, on the following search platforms: PubMed/MEDLINE, Web of Science, Scopus, LILACS, and the Cochrane Central Register of Controlled Trials. A search of the grey literature was also carried out through the Literature Report database.

[MeSH terms], keywords, and other terms used in the search are as follows: third molar [MeSH Terms], wisdom tooth [MeSH Terms], wisdom teeth [MeSH Terms], tooth extraction [MeSH Terms], surgical removal, partial odontectom *, coronectom *, nerve injury, alveolar nerve injury, mandibular nerve [MeSH Terms], inferior alveolar nerve [MeSH Terms], paresthesia [MeSH Terms], dysesthesia [MeSH Terms], lingual nerve [MeSH Terms], lingual nerve injur * [MeSH Terms]. The full search string used in databases is pointed out in Table 1.

### 2.5. Eligibility Criteria and Study Selection Process

This SR included studies that analyzed the clinical outcomes of coronectomy procedures in patients with third molars suspect to be or in contact with the IAN, without any associated pathology, with a follow-up period over 3 months. Randomized clinical trials (RCT), prospective (PS), and retrospective (RS) studies, with or without a control group (third molar extraction), were included. Case reports, in vitro studies, letters to authors, and literature reviews were excluded from the process.

The process of searching and selecting studies was conducted in duplicate by two authors (R.C.S.P. and T.C.G). The titles and abstracts were carefully evaluated according to the eligibility criteria of this SR for relevance. A consensus was established to resolve any disagreement that occurred.

### 2.6. Data Synthesis

The data from the manuscripts were extracted by R.C.S.P., in cooperation with T.C.G., and arranged as follows: author name, study design, number of coronectomy procedures, occurrence and number of IAN damage, LN damage, root migration, pain, infection, dry socket, extraction of the remaining root and the necessity for reintervention.

### 2.7. Assessment of the Risk of Bias

All eligible studies were assessed for methodological quality by two reviewers (R.C.S.P. and T.C.G). For PS, RS, and case-control articles; the quality was rated based on the Newcastle-Ottawa Quality Assessment Scale (NOS) [17]. Using the NOS, each study is judged on eight items and categorized into three groups: the selection of the study groups, the comparability of the groups, and the ascertainment of either the exposure or outcome of interest for case-control or cohort studies, respectively. RCTs involve assigning a judgment of high, low, or unclear risk of material bias for each item considered, using the Cochrane Collaboration’s tool [18] for guidance. In agreement with this, six predetermined criteria were used: sequence generation, allocation concealment, blinding, incomplete outcome data, selective outcome reporting, and other sources of bias. Material bias was then defined as a bias of sufficient magnitude to influence the results or conclusions of the trial, recognizing the subjectivity of any such judgment.

### 2.8. Statistical Analysis

The extracted data (e.g., IAN and LN damage, root migration, pain, infection, dry socket, extraction of the remaining root, and a necessity for reintervention) were pooled and analyzed qualitatively and quantitatively (variation, mean, and standard deviation) through descriptive statistics. For descriptive analysis, the StatPlus software (version 6, AnalystSoft, Walnut, CA, USA) was used. A meta-analysis pairwise cannot be performed due to the design and heterogeneity between the studies.

## 3. Results

### 3.1. Literature Search

The primary search, post the removal of duplicates, obtained 167 articles: 91 from MEDLINE/PubMed, 15 from the Cochrane Central Register of Controlled Trials, 115 from Scopus, 130 from Web of Science, 2 from LILACS, and no article in the Grey Literature Report of the New York Academy of Medicine. After the initial search, the collected data were analyzed by two independent reviewers (R.C.S.P. and T.C.G.), and the manuscripts were systematically excluded after screening titles and abstracts for relevance. Of these, 137 articles were excluded according to the exclusion criteria. Articles for which the titles and abstracts were missing sufficient information to determine eligibility according to the inclusion and exclusion criteria underwent a full-text review.

**Figure 1:**
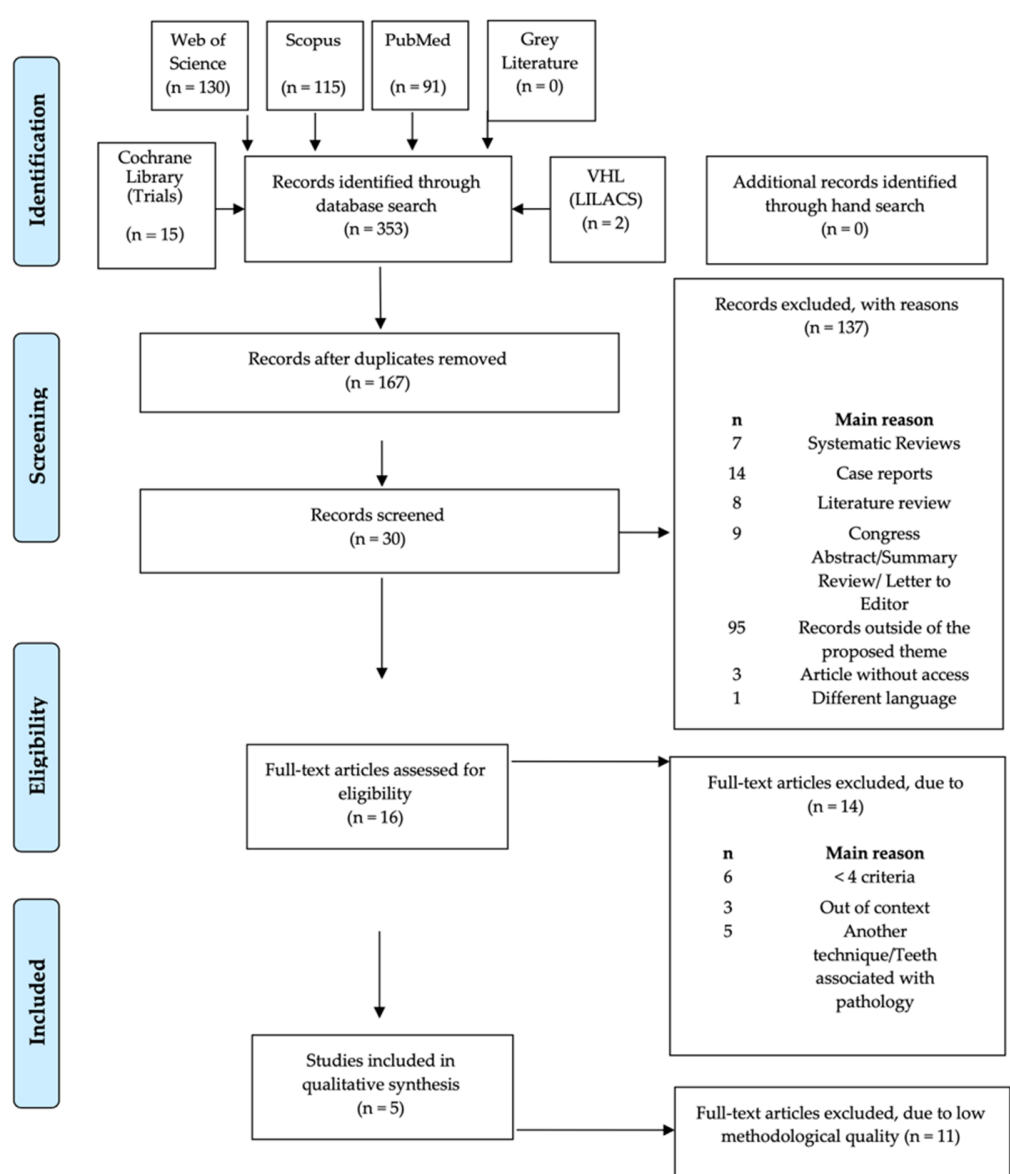
Preferred Reporting Items for Systematic Reviews and Meta-Analyses: Prisma 2009 Flow Diagram [14].

Thirty full-text records were screened for eligibility. In total, sixteen articles were included for qualitative analysis. A PRISMA diagram of the record selection process is presented in Figure 1, in which it is possible to better illustrate the search and selection process as well as the reasons given for the eliminated articles.

### 3.2. Study Characteristics

The extracted data from the studies’ compilation are presented precisely in Table 2. In total, sixteen articles were included: nine PS (Agbaje et al. [19]; Cilasun et al. [6]; Kang et al. [20]; Kowenberg et al. [3]; Leung et al. [21]; Leung et al. [22]; Mendes et al. [23]; Monaco et al. [24]; and Pedersen et al. [25]), four RS (Mukherjee et al. [26]; Frenkel et al. [2]; Kohara et al. [27]; Pogrel et al. [13]), a single study of case-control (Hatano et al. [28]) and two RCTs included studies (Leung et al. [8]; Renton et al. [29]).

Of the sixteen inserted records, all the studies presented a minimum of 20 coronectomies, a follow-up period of more than 3 months, and contain at least four of the eight evaluation criteria: IAN and LN damage, root migration, pain, infection, dry socket, extraction of the remain root and necessity for reintervention. A total of 2176 coronectomies were included, and the evaluation showed a low risk of complications after the coronectomy.

The most reported complication was pain in 22.04% of the procedures. The IAN injury was documented in 0.59% of the proceedings, LN injury in 0.22%, infection 3.95%, dry socket 1.12%, extraction of the root 5.28%, and reintervention necessity 1.13%.

#### 3.2.1. IAN Injury

Eight authors (Frenkel et al. [2], Hatano et al. [28], Kohara et al. [27], Leung et al. [8], Leung et al. [22], Leung et al. [21], Mendes et al. [23] and Pedersen et al. [25]) reported the occurrence of IAN damage post coronectomy procedure. Mendes et al. [23] presented 2.85% (one case) and Pedersen et al. [25] 2.16% (five cases) differently to the average, which was 0.59%. All the other articles cited that no patient experienced numbness, swelling or a thickening sensation of the lower lips after surgery after the procedure.

#### 3.2.2. LN Injury

Compared with the IAN damage (total of thirteen cases), the LN injury (four cases) was less common. Of the sixteen studies, only three (Mukherjee et al. [26], Pedersen et al. [25], and Pogrel et al. [13]) reported the occurrence of this neurological alteration.

Four articles (Frenkel et al. [2], Kang et al. [20], Kohara et al. [27], and Mendes et al. [23]) provided no records or insufficient data concerning the sensitivity of the lingual nerve postsurgical procedures.

All of the remaining articles assessed no occurrence of damage on the lingual nerve following coronectomy/complete extraction.

#### 3.2.3. Root Migration

Eleven authors (Agbaje et al. [19]; Kang et al. [20]; Hatano et al. [28]; Kohara et al. [27]; Kouwenberg et al. [3]; Leung et al. [8]; Leung et al. [22]; Mendes et al. [23]; Mukherjee et al. [26]; Pogrel et al. [13]; Renton et al. [29]) recorded the occurrence of root migration. 

The high incidence (more than 80% of their cases) of root migration was reported in Kang et al. [20]; Hatano et al. [28]; Kohara et al. [27]; Kouwenberg et al. [3]; Mendes et al. [23]. Coronal migration of the roots was reported as the most commonly reported long-term consequence of coronectomy, which is confirmed by our review.

#### 3.2.4. Pain

The onset of pain was evaluated in eleven of the included articles (Agbaje et al. [19]; Cilasun et al. [6]; Frenkel et al. [2]; Hatano et al. [28]; Leung et al. [8]; Leung et al. [22]; Leung et al. [21]; Mendes et al. [23]; Mukherjee et al. [26]; Pedersen et al. [25]; Renton et al. [29]).

The assessment of pain with a Visual Analog Scale (VAS) post coronectomy was present in Mendes et al. [23] and Mukherjee et al. [26]. Both authors analyzed the first week after the procedure, but Mukherjee et al. [26] cited a case of one patient that reported discomfort in mouth-opening for a period of two months, which was attributed to enamel lipping left behind intraoperatively, and subsided spontaneously. The other studies evaluated pain based on the patient’s complaint.

#### 3.2.5. Infection

Comparing the quantity of all the procedures performed by the authors that analyzed these criteria (Agbaje et al. [19]; Cilasun et al. [6]; Frenkel et al. [2]; Kang et al. [20]; Hatano et al. [28]; Leung et al. [8]; Leung et al. [22]; Leung et al. [21]; Mendes et al. [23]; Monaco et al. [24]; Pedersen et al. [25]; Renton et al. [29]) the percentage of infection was 3.95% (73 cases), which was higher in Pedersen et al. [25], with 27 cases (11.68%), more than one-third of the total cases. The authors of this study justified this expressive rate as being similar to corresponding studies after both coronectomy and total removal of mandibular third molars. Another hypothesis is that the indication for the total removal of these teeth was adequate.

In sequence, Leung et al. [8] with 5.26%, Renton et al. [29] with 5.17%, and Leung et al. [22], all had 4.4%. Two articles, Kang et al. [20] and Monaco et al. [24], did not present any case of infection, and the time of follow-up for them was, respectively, three years and five years. Furthermore, three articles did not discuss these criteria: Kohara et al. [27], Kouwenburg et al. [3], and Pogrel et al. [13].

#### 3.2.6. Dry Socket

The incidence of the early complication included in this study was 16 cases (1.12%) of all articles that appraised this criterion (1428 coronectomies). In Cilasiun et al. [6], Leung et al. [8,22], and Pederson et al. [25], there were no cases of dry sockets. In the randomized study of Renton et al. [29], the percentage of dry sockets was 12.06%, the highest of all, followed by Agbaje et al. [19], with 4.16%.

Kang et al. [20], Kouwenberg et al. [3], Mendes et al. [23], Monaco et al. [24], Mukherjee et al. [26], Pederson et al. [25], Pogrel et al. [13] did not evaluate these criteria during their study.

#### 3.2.7. Extraction of the Remaining Root

The necessity of extraction of the remaining root occurred in all studies. This later complication occurred in 115 cases (5.28%) of all the 2176 coronectomies. Discriminating the quantity of extraction of the remaining roots, Kouwenberg et al. [3] had almost a triple percentage of this complication (11.26%), followed by Mukherjee et al. [26] with 10%, Agbaje et al. [19] with 9.37% and Leung et al. [22] with 9.35%.

The study conducted by Mukherjee et al. [26] had two patients with failed coronectomy, with the mobilized roots removed. Both cases were female patients with conical root morphology.

#### 3.2.8. Necessity for Reintervention

The necessity for reintervention excluded the extraction of the remaining root, with only the incomplete removal of the crown or enamel retention and the exposure of the root in the oral cavity. This late complication is related to root migration. Only six articles assessed these criteria.

Comparing the number of coronectomies that evaluated these criteria, the percentage of this complication was 1.13% (12 cases), lower than Frenkel et al. [2] and Monaco et al. [24], who each presented four cases, two-thirds of the cases analyzed. Cilasun et al. [6] and Renton et al. [29] described any case of reintervention during their period of follow-up, of seventeen and thirteen months, respectively.

### 3.3. Assessments of the Risk of Bias

NOS for cohort and case-control was used for a critical appraisal (presented in Table 3). In total, fourteen articles were evaluated, including PS (Agbaje et al. [19]; Cilasun et al. [6]; Kang et al. [20]; Kouwenberg et al. [3]; Leung et al. [21]; Leung et al. [22]; Mendes et al. [23]; Monaco et al. [24] and Pedersen et al. [25]), four RS (Mukherjee et al. [26]; Frenkel et al. [2]; Kohara et al. [27]; Pogrel et al. [13]), and one case-control study (Hatano et al. [7]).

According to this methodological quality assessment, most of the articles were poorly judged, scoring 3 of 9 points for overall NOS (Frenkel et al. [2]; Kowenberg et al. [3]; Leung et al. [21]; Mendes et al. [23]; Mukherjee et al. [26]; Pogrel et al. [13]). Four of them scored 4 (Agbaje et al. [19]; Kohara et al. [27]; Leung et al. [22]; Monaco et al. [24]; Pedersen et al. [25]), one of the studies scored a 6 (Cilasun et al. [6]), another scored 7 (Kang et al. [20]), and only one of the papers presented a high NOS value, a score of 8 (i.e., low risk of bias).

**Figure 2:**
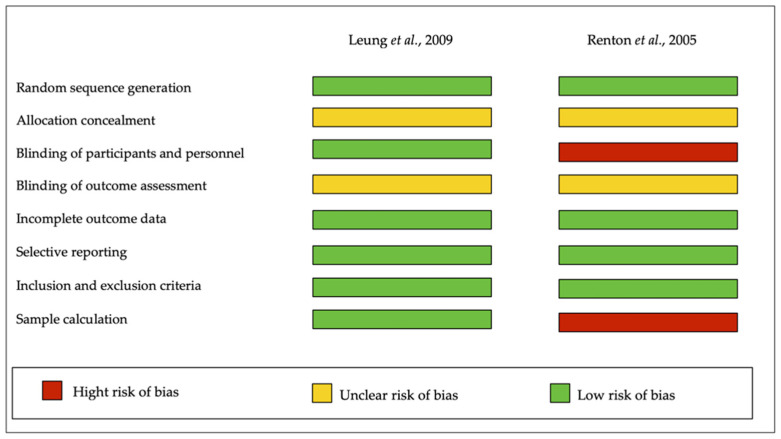
Cochrane Collaboration`s tool for assessing Risk of Bias.

Diversely, for randomized clinical trials, the Cochrane Collaboration’s tool was used. Figure 2 showed that the two included studies (Leung et al. [8]; Renton et al. [29]). Leung et al. [8] were best ranked, exhibiting a low risk of bias in the majority of indicators.

## 4. Discussion

This SR evaluated the rate of complications to analyze the success of coronectomy. This procedure is indicated in inferior third molars with proximity to or real contact with IAN. However, the coronectomy has other benefits beyond IAN preservation, such as avoiding LN injury and a less morbid procedure. For this, migration of the root that minimizes damage to the IAN with the extraction of the remaining root is necessary.

The higher limitation of the study was due to the heterogeneity of the studies included (PS, case-control, and RCT), making it necessary to perform a descriptive analysis of the eight criteria. The evaluation of the PS and RS studies using the NOS assessment demonstrated that only three (Hatano et al. [28]; Kang et al. [20] and Cilasiun et al. [6]) of the fourteen articles achieved a good methodological quality assessment. Additionally, the randomized studies (Renton et al. [29] and Leung et al. [8]) presented good qualifications. Due to the design (low number of clinical trials) and heterogeneity of the studies, it was not possible to carry out the meta-analysis pairwise.

In this SR, the IAN injury results were minimal, almost 0.6%, endorsing the idea of this being a good indication of technique for the extraction of third molars in proximity to the IAN, compared with the extraction of 18.6%, as cited in the Renton et al. [29]. Furthermore, the literature advises that there is a low possibility of LN injury, and our findings indicated the same, with injury being temporary and self-resolving.

The comparison of the loss of sensitivity between full extraction and coronectomy of the lower third molar was evaluated by Hatano et al. [28]. The extraction group consisted of six patients (5%) with signs of IAN injury (three of them were permanent). In contrast, in the coronectomy group, one patient (1%) complained of nerve symptoms postoperatively, but became asymptomatic within 1 month. Leung et al. [8] showed a postoperative neurosensory deficit in one patient (0.65%) of the coronectomy group, whereas nine cases (5.10%) occurred after the complete extraction. The findings of these studies corroborate the idea that coronectomy is a valid procedure to minimize the possibility of IAN damage.

The incidence of IAN injury post coronectomy in Frenkel et al. [2] was 0.5% (1 of 185 patients). This injury was temporary and manifested as hypoesthesia of the lower lip. Frenkel et al. [2], Kohara et al. [27] reported one hypoesthesia subject (1%) on a postoperative day, associated with surgical difficulties, which improved after 2 months. 

The pattern of root migration is well-described in Leung et al. [30], showing that this migration is common in the first two years after coronectomy, and decreases after this period.

One of the studies [16] reported that 97% of the retained roots showed signs of migration, and 65% showed signs of rotation. Panoramic radiograph analysis showed that migration mainly occurred during the first year and that this migration led to a structural separation between the root complex and the mandibular canal in more than half of the cases. In addition, Kohara et al. [27] investigated the migration pattern of the retained roots postoperatively after 3 years. In the first year, a rate of 74.3% root migration was found. The study showed that root migration increased in the first 2 years after coronectomy, but stabilized between the second and the third year.

Our study evaluated whether this movement were present or not, and we found that half the cases had some degree of migration (50%). The most critical evaluation that those articles discussed was that the migration of the roots was moving away from the IAN, and some of them could expose of the root to the oral cavity, necessitating surgical reintervention for enamel edge trimming or extraction of the remaining root. All the articles included in this evaluation stated that this is a regular complication, and that it is essential to explain the possible necessity of a new procedure to the patient. The period of stabilization was similar to the one cited in Leung´s article: approximately three years.

The principal, short-term complications that can be found after the coronectomy procedure are pain, infection, and dry socket. These complications may occur in the same proportion during tooth extraction [29].

The assessment of pain with the Visual Analog Scale (VAS) post coronectomy by Mukherjee et al. [26] showed that three (out of a total of 20) coronectomy sites developed pain after the first week of the procedure. One patient had pain on the soft tissue around the coronectomy site, with discomfort in mouth-opening for two months. This was attributed to enamel lipping left behind intraoperatively, and subsided spontaneously. 

According to Leung et al. [8], among the coronectomy group, 41.9% of the teeth were reported to be in pain 1 week postoperatively. The corresponding proportion in the control group was 57.3%, which was statistically significant. However, there was no difference between the groups from 1 to 24 months after surgery.

Only one patient presented pain (1.13%) following the coronectomy, with no pain reported for the coronectomy group, according to Cilasun et al. [6]. These results cause a discrepancy with Cilasun et al. [6], Hatano et al. [28], who estimated a relevant difference in pain measures, with the incidence of postoperative pain being more significant in the coronectomy group. In the coronectomy group, 18.63% showed pain, compared to 6.78% in the extraction group; however, all pain decreased within 1 week. This information assists the professional in choosing the appropriate pain control medications. Renton et al. [29] considered that the selection of patients with a high proportion of difficult and deeply impacted teeth could be the reason for the higher rate of dry sockets. Agbaje et al. [19] were the only authors that described how their group managed the dry socket. The procedure consisted of wound debridement and irrigation, after which Alvogyl™ (Septodont; Saint-Maur-des-Fossés, France) paste-dressing material was placed to control pain: an effective treatment which led to appropriate healing.

Favorable factors to predict the failure of coronectomy, leading to a second surgical approach to the root extraction, may occur most often in women with conically rooted teeth that narrow within the nerve canal [29].

## 5. Conclusions

Coronectomy may be considered a low-risk procedure, and an option for treatment to avoid potentially more severe damage to nervous structures. The reintervention to remove the remaining roots or the reduction in the remaining roots may be considered part of the treatment because the possibility of this causing nerve injury reduces, due to the migration of the roots. This is the main reason to select coronectomy over the extraction of the third molar, close to or in contact with the IAN. However, patients should still undergo a full screening and evaluation of postsurgical procedures.

## Figures and Tables

**Table 1:** The electronic database used and search strategy.

Database	Search Strategy
**PubMed**	#1 ((((((((third molar * [MeSH Terms]) OR third molar * [Title/Abstract]) OR wisdom tooth [MeSH Terms]) OR wisdom tooth [Title/Abstract]) OR wisdom teeth [MeSH Terms]) OR wisdom teeth [Title/Abstract]) OR tooth extraction * [MeSH Terms]) OR tooth extraction * [Title/Abstract]) OR surgical removal [Title/Abstract] #2 (partial odontectom * [Title/Abstract]) OR coronectom * #3 (((((((((((((nerve injury [Title/Abstract]) OR alveolar nerve injury [Title/Abstract]) OR mandibular nerve * [MeSH Terms]) OR mandibular nerve * [Title/Abstract]) OR Inferior Alveolar Nerve * [MeSH Terms]) OR Inferior Alveolar Nerve * [Title/Abstract]) OR paresthesia * [MeSH Terms]) OR paresthesia * [Title/Abstract]) OR dysesthesia * [MeSH Terms]) OR dysesthesia * [Title/Abstract]) OR lingual nerve * [MeSH Terms]) OR lingual nerve * [Title/Abstract]) OR lingual nerve injur * [MeSH Terms]) OR lingual nerve injur * [Title/Abstract] #1 and #2 and #3
**Web of Science**	#1 TOPIC: (third molar *) OR TOPIC: (wisdom tooth) OR TOPIC: (wisdom teeth) OR TOPIC: (tooth extraction *) OR TOPIC: (surgical removal) #2 TOPIC: (partial odontectom *) OR TOPIC: (coronectom *) #3 TOPIC: (nerve injury) OR TOPIC: (alveolar nerve injury) OR TOPIC: (mandibular nerve *) OR TOPIC: (inferior alveolar nerve *) OR TOPIC: (paresthesia *) OR TOPIC: (dysesthesia *) OR TOPIC: (lingual nerve *) OR TOPIC: (lingual nerve injur *) #1 and #2 and #3
**Scopus**	#1 Topic: (TITLE-ABS-KEY (third AND molar *) OR TITLE-ABS-KEY (wisdom AND tooth) OR TITLE-ABS-KEY (wisdom AND teeth) OR TITLE-ABS-KEY (tooth AND extraction *) OR TITLE-ABS-KEY (surgical AND removal)) #2 Topic: (TITLE-ABS-KEY (partial AND odontectom *) OR TITLE-ABS-KEY (coronectom *)) #3 Topic: (TITLE-ABS-KEY (nerve AND injury) OR TITLE-ABS-KEY (alveolar AND nerve AND injury) OR TITLE-ABS-KEY (mandibular AND nerve *) OR TITLE-ABS-KEY (inferior AND alveolar AND nerve *) OR TITLE-ABS-KEY (paresthesia *) OR TITLE-ABS-KEY (dysesthesia *) OR TITLE-ABS-KEY (lingual AND nerve *) OR TITLE-ABS-KEY (lingual AND nerve AND injur *)) #1 and #2 and #3
**Cochrane Library**	third molar * OR wisdom tooth OR wisdom teeth OR tooth extraction * OR surgical removal in Title, Abstract, Keywords, and partial odontectom * OR coronectom * in Title, Abstract, Keywords and nerve injury OR alveolar nerve injury OR mandibular nerve * OR inferior alveolar nerve * OR paresthesia * OR dysesthesia * OR lingual nerve * OR lingual nerve injur * in Title, Abstract, Keywords in Trials
**VHL** **(LILACS)**	(tw:(third molar OR tooth extraction)) AND (tw:(partial odontectom * OR coronectom *)) AND (tw:(mandibular nerve OR paresthesia OR lingual nerve *)) AND (instance:”regional”) AND (db:(“LILACS”))

**Table 2:** Corononectomies evaluation included in each study.

Author	Included Coronectomies (n)	IAN Damage (%)	LN Damage (%)	Pain (%)	Infection (%)	Dry Socket (%)	Extraction of the Root (%)	Surgical Reintervention (%)
Agbaje et al., 2015	96	0%	0%	4.16%	4.16%	4.16%	9.37%	*
Cilasun et al., 2011	88	0%	0%	1,13%	1.13%	0%	1.13%	0%
Frenkel et al., 2015	185	0.54%	*	8.65%	1.62%	*	3.24%	2.16%
Hatano et al., 2009	102	0.98%	0%	18.63%	0.98%	1.96%	4.9%	0.00%
Kang, 2019	55	0%	*	*	0%	2%	9.09%	*
Kohara et al., 2015	111	0.9%	*	*	*	0.9%	9.01%	*
Kouwenberg et al., 2016	151	0%	0%	*	*	*	11.26%	*
Leung et al., 2009	171	1.17%	0%	38.01%	5.26%	0%	9.36%	*
Leung et al., 2012	135	0.74%	0%	42.96%	4.44%	0%	2.96%	*
Leung et al., 2016	612	0.16%	0%	31.21%	2.94%	0.16%	2.94%	0.33%
Mendes, 2020	35	2.85%	*	48.57%	2.85%	*	8.57%	5.71%
Monaco, 2019	76	0%	0%	*	0%	*	6.57%	5.26%
Mukherjee et al., 2016	20	0%	5%	15%	*	*	10%	*
Pederson et al., 2018	231	2.16%	0.86%	0%	11.69%	*	3.46%	*
Pogrel et al., 2004	50	0%	2%	*	*	*	6%	*
Renton T et al., 2005	58	0%	0%	13.79%	5.17%	12.06%	8%	0%
Total	2176							

* No available data.

**Table 3:** Newcastle-Ottawa Quality Assessment Scale (NOS) Cohort and Case-control studies.

Author, Year	Selection				Comparability	Outcome			
	Representativeness of the Exposed Cohort	SELECTION of External Control	Ascertainment of Exposure	Outcome of Interest Not Present at the Start	Comparability of Cohorts on the Basis of the Design of Analysis	Assessment of the Outcome	Was Follow-up Long Enough for Outcomes Occur	Adequacy of Follow up of Cohorts	Total 9/9
Agbaje et al., 2015	0	0	*	0	0	*	*	*	4/9
Cilasun et al., 2011	0	*	*	0	**	*	*	0	6/9
Frenkel et al., 2015	0	0	*	0	0	*	*	0	3/9
Kohara et al., 2015	0	0	*	0	0	*	*	*	4/9
Kouwenberg et al., 2016	0	0	*	0	0	*	0	*	3/9
Leung et al., 2012	0	0	*	0	0	*	*	*	4/9
Leung et al., 2016	0	0	*	0	0	*	*	0	3/9
Mukherjee et al., 2016	0	0	*	0	0	*	*	0	3/9
Pogrel et al., 2004	0	0	*	0	0	*	0	*	3/9
Pedersen et al., 2018	0	0	*	0	0	*	*	*	4/9

A study can be awarded a maximum of one star (*) for each item within the Selection and Outcome/Exposure categories. A maximum of two stars (**) can be given for comparability.

## Data Availability

Not applicable.
